# A systematic literature review on the European, African and Amerindian genetic ancestry components on Brazilian health outcomes

**DOI:** 10.1038/s41598-019-45081-7

**Published:** 2019-06-20

**Authors:** Fabiana dos Santos Carolino Firmo Pereira, Raphael Mendonça Guimarães, Alexandre Ramos Lucidi, Doralina Guimarães Brum, Carmen Lucia Antão Paiva, Regina Maria Papais Alvarenga

**Affiliations:** 1 Department of Neurology, Graduate Program in Neurology (PPGNEURO) – Universidade Federal do Estado do Rio de Janeiro, UNIRIO, Rio de Janeiro, RJ 20270-004 Brazil; 2 Research Center for Population and Public Policies Studies, Rio de Janeiro, Brazil. Oswaldo Cruz Foundation, Rio de Janeiro, RJ 21040-900 Brazil; 3 Departamento de Neurologia, Psicologia e Psiquiatria, Faculdade de Medicina de Botucatu, Universidade Estadual Paulista -UNESP, Botucatu, São Paulo Brazil; 4 Department of Genetics and Molecular Biology, UNIRIO, Rio de Janeiro, 20211-010 RJ Brazil; 5 Graduate Program in Molecular and Cell Biology (PPGBMC). UNIRIO, Rio de Janeiro, RJ 20211-010 Brazil

**Keywords:** Genetics research, Genetics research, Genetic markers, Genetic markers, Genetic markers

## Abstract

The variables such as race, skin colour and ethnicity have become intensely discussed in medicine research, as a response to the rising debate over the importance of the ethnic-racial dimension in the scope of health-disease processes. The aim of this study was to identify the European (EUR), African (AFR) and Amerindian (AMR) ancestries on Brazilian health outcomes through a systematic literature review. This study was carried out by searching in three electronic databases, for studies published between 2005 and 2017. A total of 13 papers were eligible. The search identified the following health outcomes: visceral leishmaniosis, malaria, Alzheimer’s disease, neuromyelitis optica, multiple sclerosis, prostate cancer, non-syndromic cleft lip/palate, chronic heart failure, sickle cell disease, primary congenital glaucoma, preterm labour, preterm premature rupture of membranes, systemic lupus erythematosus and type 1 diabetes mellitus. Research paper assessments were guided by the STROBE instrument, and agreements between results were determined by comparing the points attributed by two authors. Increased EUR ancestry was identified from preterm labour (PTL), type 1 diabetes (T1D) and non-syndromic cleft lip with or without cleft palate (NSCL), as well as in patients presenting aggressive prostate cancer prognoses. On the other hand, the highest AFR ancestral component was verified from systemic lupus erythematosus (SLE) and primary congenital glaucoma (PCG) cases, presenting worse prognoses. AMR ancestry may be a protective factor in the development of Alzheimer’s disease (AD). The worst hemodynamic parameters in cases of heart failure (HF) were identified among individuals with greater AMR and AFR ancestry indices.

## Introduction

Over 9.5 million Africans were brought to the Americas between the 15^th^ and 19^th^ centuries, with 38% of this total arriving in Brazil^1^. The main destinations in the country were the most important cities at the time: Salvador, Recife and Rio de Janeiro^2^. In the 19^th^ century, new immigrants arrived at this colony from Italy, Spain and Germany, as well as a lower amount of Japanese immigrants (about 90% of this particular population settled in the state of São Paulo) and also Syrians and Lebanese. Brazil was perceived as a country of many opportunities in Europe and Asia (especially Japan)^3^. Between 1872 and 1890, the Brazilian population increased by 12.5 million, with an excess amount of men in the groups, thus leading to inter-ethnic relations among European men and African or Amerindian women. This tri-hibrid composition found in the Brazilian population, comprising Amerindians (AMR), Europeans (EUR) and Africans (AFR), was historically described by the Brazilian anthropologist Darcy Ribeiro, in “The Brazilian People”^4^. This miscegenation process led to sub-populations with differences in the proportion of admixed individuals.

Clinical epidemiology seeks valid conclusions to better define health policies, predictions concerning risk (or protection) and individual prognoses within each population group. It is common that the chosen research design be case-control studies, which present scientific relevance and feasibility. However, the greatest weakness of this design is a significant susceptibility to selection and measurement biases, with emphasis on calibration bias^5^, especially when qualitatively measured attributes are subjective, such as “race/colour”, varying according to circumstances and context^6^. On the other hand, the issue of undetected misleading effects is noted when the investigated population is composed of subpopulations presenting several ancestry backgrounds (with different allelic frequencies and risk for disease occurrence). This leads to unequal representation in case and control groups, which may result in false-positive associations during statistical analyses^7–9^. Thus, additional methods that take into account local ancestry in predicting genetic risk are required, as they incorporate different ancestry linkage imbalances and their own allelic frequencies^10,11^. Because of this, spurious associations unrelated to causative *loci* can be found in population substructures present in larger investigated populations that cannot be identified in isolation^12^.

Currently, the word “race” can be used in many ways, including the morphological, phenotypic sense, denoting a set of physical characteristics (e.g. skin colour or hair texture), which allows for the identification of individuals as belonging to a certain group^13^. In the last three decades, race, colour and ethnicity have become intensely discussed in collective health and medicine studies, as a response to the rising debates concerning the importance of the ethnic-racial dimension in health-disease processes^10,14,15^. Since 1998, Brazilian health information systems have necessarily included the variable “race/skin colour”, according to the five categories described by the Brazilian Institute of Geography and Statistics^16^. It should be noted that, due to the differences in racial categorization methodologies among the selected papers, the authors were not able to summarize, in this recent systematic review, case-by-case disease prevalence estimates according to race/skin colour^17^.

EUR, AFR and AMR genetic ancestry can be estimated quantitatively through the use of ancestral informational markers (AIMs). The AIMS can be classified as: short tandem repeats (STR) autosomal haplotypes, insertion/deletion (INDEL) and single nucleotide polymorphisms (SNP) haplotypes which, due to extensive recombination, are evanescent, constituting excellent markers for individuality. Other markers such as uniparental maternal mitochondrial DNA (mtDNA) and paternal non-recombinant Y-chromosome polymorphic region (NRY), are excellent lineage markers, they are haploid and do not undergo recombination^18^. Regarding the number of markers, different sampling strategies can be applied, and all can contribute to the final results when genetic ancestry^19–22^ is assessed. A meta-analysis, published in 2015 identified the genetic miscegenation of the Brazilian people, with the following ancestry components proportions: EUR 0.62, AFR 0.21 and AMR 0.17. The authors identified that Brazil presents the 5^th^ greatest ancestral EUR proportion in Latin America, the 12^th^ AFR proportion and the 10^th^ largest AMR ancestry^19,23^. These results contrast with the last Brazilian census results regarding skin colour, carried out in 2010 by the IBGE, in which a qualitative perception was noted that the white population no longer comprised the majority of Brazilians. In the same year, 47.7% of the interviewed Brazilians described themselves as white, 43.1% as brown, 7.6% as black, 1.1% as yellow and 0.4% as indigenous^16^. Brazilian systematic review pointed out that race does not have a biological relationship with health^17^. In this scenario, a systematic literature review of the European, African and Amerindian ancestry components on Brazilian health outcomes was carried out. However, the reader should be aware that it was not our objective to report the heritability value (h2) for each disease discussed here, since none of the 13 Brazilian articles eligible for this review presented these values; although heritability is a parameter that can help understand the genetic architecture of complex traits.

## Results

**Figure 1:**
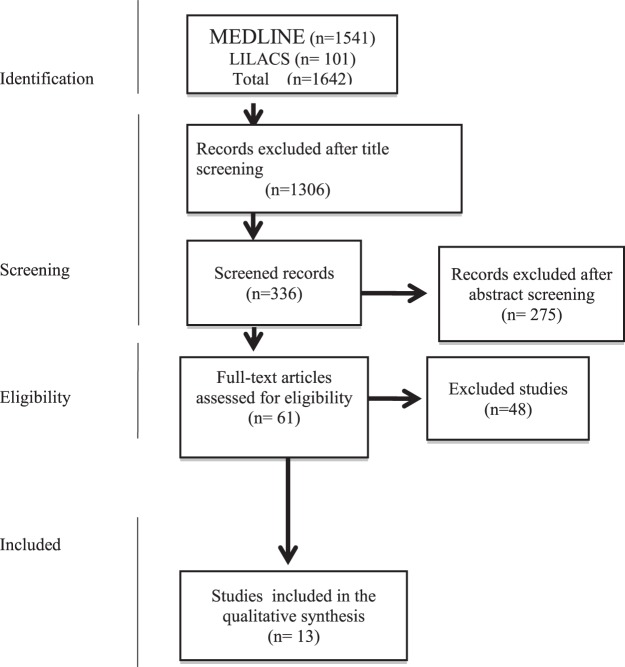
Study identification flowchart. MEDLINE = Medical Literature Analysis and Retrieval System Online; LILACS = Scientific and Technical Literature of Latin America and the Caribbean/VHL - Virtual Health Library

This study aimed to carry out a systematic literature review on the EUR, AFR and AMR ancestry components on health outcomes in Brazil, i.e., identifying the three main ancestry components that could contribute to Brazilian people health outcomes. The search identified the following health outcomes: visceral leishmaniosis (VL), malaria, Alzheimer’s disease (AD), neuromyelitisoptica (NMO), multiple sclerosis (MS), prostate cancer (PCa), non-syndromicoral clefts, chronic heart failure (HF), sickle cell disease (SCD), primary congenital glaucoma (PCG), preterm labour (PTL), preterm premature rupture of membranes (PPROM), systemic lupus erythematosus (SLE) and type 1 diabetes mellitus (TD1) (Table 1). Thirteen studies met previously set criteria. No studies featuring isolated Brazilian populations were selected. The general characteristics of the selected paper are listed in Table 1^24–36^. The electronic search comprised only studies published between 2005 and 2017. A total of 1642 published studies fitting the study criteria were identified at the searched databases (Fig. 1). The scientific journals Annals Human Genetic, Plos One and Genetics and Molecular Research presented citation frequencies of 2/13 each. Dementia Geriatric Cognitive Disorders, Journal Oral Pathology Medicine, Open Heart, Journal Glaucoma, Mediators of Inflammation and Scientific Reports and BMC Pregnancy and Childbirth, appeared only once (n = 13). More than half (53.84%) of the publications were from 2016. Samples from the five Brazilian regions (North Region, Southeast Region, Northeast Region and South Region and Center-West Region) are represented in the selected papers, as displayed in Table 2.

**Table 1:** General characteristics of the selected papers.

Author	Disease	Scientific Journal	Study design	Sample	European percentage	African percentage	Amerindian percentage
Ettinger *et al*.^28^	Visceral Leishmaniosis	Ann Hum Genet	transversal	439	**47%**	29%	24%
Tarazona-Santos *et al*.^29^	Malaria	Plos One	case-control	282	**54%**	18%	28%
Benedet *et al*.^26^	Alzheimer’s disease	Dementia Geriatric Cognitive Disorders	coorte	532	**56.80%**	29.30%	13.90%
Brum *et al*.^25^	NMO^a^ and MS^b^	Plos One	case-control	344	**NMO 68.7%** **MS 78.5%**	NMO 20.5% MS 12.5%	NMO 10.8% MS 9%
Oliveira *et al*.^30^	Prostate Cancer	Genetics and Molecular Research	case-control	213	**case 46%** control 61%	case 44% control 33%	case 10% control 6%
Messetti *et al*.^35^	Non-syndromic cleft lip	Journal Oral Pathol Med	case-control	1478	**NSCL**^**c**^ **± P 82.0%** **NSCPO**^**d**^ **76.4%** Control 84.3%	NSCL ± P 16.2% NSCPO 21.4% Control 14.0%	NSCL ± P 1.8% NSCPO 2.2% Control 1.7%
Bernardez-Pereira *et al*.^31^	Chronic heart failure	Open Heart	transversal	362	General 61% **White 80%** Brown 58% Black 32%	General 29% White12% Brown 31% **Black 61%**	General 6% White 8% Brown 11% Black 7%
Nascimento *et al*.^32^	Sickle cell disease	Genetics and Molecular Research	transversal	20	**44%**	42%	11%
Rolim *et al*.^33^	PCG^e^	Journal Glaucoma	case-control	90	**case 78.4%** control 73.0%	case 14.9% control 13.2%	case 6.7% control 13.8%
Ramos *et al*.^36^	PTL^f^ and PPROM^g^	BMC Pregnancy and Childbirth	case-control	735	**PTL 70.5% PPROM 67.7% control 64.4%**	PTL 14.1% PPROM 15.1% control 17.8%	PTL 12.1% PPROM 12.8% control 11.7%
Furini*et al*.^27^	Malaria *Vivax*	Mediators Of Inflammation	case-control	141	**Case 44.2%** control 44.9%	case 31.8% control 29.5%	case 24% control 25.6%
Barbosa *et al*.^24^	SLE^h^	Annals Of Human Genetics	case-control	133	**SNPs**^**i**^**66.21%AIMs**^**j**^**65.56%**	SNPs 21.97% AIMs 20.97%	SNPs 11.82% AIMs 13.45%
Gomes *et al*.^34^	T1D^l^	Scientific Reports	case-control	1704	**Case 77%** control 71%	Case 15% control 21%	Case 7.3% control 7.9%

^a^Neuromyelitis optica; ^b^Multiple sclerosis; ^c^Non-syndromic cleft lip with or without cleft palate; ^d^Non-syndromic cleft palate only; ^e^Primary congenital glaucoma; ^f^Preterm Labor; ^g^Preterm Premature Rupture of Membranes; ^h^Systemic lupus erythematosus; ^i^Single nucleotide polymorphisms; ^j^Ancestry informative markers; ^l^Type 1 diabetes mellitus; The most frequent ancestry component is marked in bold.

**Table 2:** Samples from the five Brazilian regions.

Year	Study Identification	N. of Participants	Disease	Brazilian Region	Type of markers	N. of markers
2009	Ettinger *et al*.^28^	439	Visceral Leishmaniose	North	STRs^a^	289
2011	Tarazona-Santos *et al*.^29^	282	Malaria	North	SNPs^b^ INDELs^c^	14 48
2012	Benedet *et al*.^26^	532	Alzheimer’s disease	Center-West	SNPs	12
2013	Brum *et al*.^25^	344	NMO^d^ and MS^e^	Southeast Center-West Northeast	SNPs INDELs Alu	12
2016	Oliveira *et al*.^30^	213	Prostate Cancer	Northeast	SNPs INDELs	11
2016	Messetti *et al*.^35^	1478	Non-syndromic cleft lip	South Northeast	INDELs	40
2016	Bernardez-Pereira *et al*.^31^	362	Chronic heart failure	Southeast	SNPs	101 348
2016	Nascimento *et al*.^32^	20	Sickle cell disease	Northeast	SNPs	8
2016	Rolim *et al*.^33^	90	PCG^f^	Southeast	INDELs	40
2016	Ramos *et al*.^36^	414	PTL^g^ and PPROM^h^	Southeast	INDELs	61
2016	Furini *et al*.^27^	141	Malaria *Vivax*	North	INDELs	48
2017	Barbosa *et al*.^24^	133	SLE^i^	Southeast	SNPs; AIMs^j^ array	345 12
2017	Gomes *et al*.^34^	1704	T1D^l^	Southeast	SNPs	89

^a^Short tandem repeats; ^b^Single nucleotide polymorphisms; ^c^Insertion/deletions; ^d^Neuromyelitis optica; ^e^Multiple sclerosis; ^f^Primary congenital glaucoma; ^g^Preterm Labor; ^h^Preterm Premature Rupture of Membranes; ^i^Systemic lupus erythematosus; ^j^Ancestry-informative-marker; ^l^Type1 diabetes mellitus.

The selected papers were produced by groups with different health sciences specializations. All nine clinical specialties discussed ancestry genetic components: three of the 13 studies discussed infectology^27–29^, and two are neurology papers^25,26^. Publications at 1/13 data were autoimmune disease^24^, urology^30^, cardiology^31^, hematology^32^, ophthalmology^33,34^, bucomaxillae^35^, obstetrics^36^. The case-control study design^24,25,27,29,30,33–36^, represented 69.23% of the studies analysed in this review. Cross-sectional designs^28,31,32^ and longitudinal studies^26^ accounted for 23.07% and 7.69%, respectively. It should be noted that 30.76% of the selected studies were pioneer investigations in applying the AIM methodology for Brazilian populations, comprising the following outcomes: HF, a major morbidity and mortality cause worldwide, with a significant impact on health systems^31^; NMO and MS, rare demyelinating neurological diseases affecting the central nervous system^25^; PTL and PPROM, which contribute significantly to neonatal mortality and morbidity worldwide^36^, and primary PCG, a rare disease which is a major cause of blindness^33^.

The AIMs used in the ancestry component analyses between cases and controls were categorized into three types: INDELs, SNPs and STRs. Each research group decided whether to use one or more AIMs. Some analyses used two to three types of markers, i.e. 30.76% of the studies, in malaria^29^, NMO and MS cases^25^, and for patients presenting PCa^30^ and SLE^24^. The other studies chose either INDELs^27,33,35,36^; SNPs^26,31,32,34^ or STRs^28^. No study applied mtDNA or NRY markers to investigate ancestry contributions.

Increased EUR ancestry was identified from preterm labour (PTL), type 1 diabetes (T1D) and non-syndromic cleft lip with or without cleft palate (NSCL), as well as in patients presenting aggressive prostate cancer prognoses. On the other hand, the highest AFR ancestral component was verified from systemic lupus erythematosus (SLE) and primary congenital glaucoma (PCG) cases, presenting worse prognoses. AMR ancestry may be a protective factor in the development of Alzheimer’s disease (AD). The worst hemodynamic parameters in cases of heart failure (HF) were identified among individuals with greater AMR and AFR ancestry indices (Table 1).

## Discussion

Sociological and anthropological methodologies were, for decades, tools applied for the analysis of Brazilian origins^4^. Spurious associations unrelated to causative *loci* can be obtained when the subpopulation structure is not measured. The unique characterization of population substructure genetic ancestries avoids possible confusion concerning Brazilian population samples^24^.

The ideal of building a nation modelled after Europe, throughout the 19^th^ century, made it difficult for Asian workers to immigrate to Brazil. The Brazilian upper classes debated on the ideology of skin whitening and the endeavour of making Brazil a “civilized” country, leading to race barriers for immigrants^3^. This is why two of the selected papers chose to describe the exclusion of Asian descendant patients from the analyses^25,26^.

A new and valuable insight into the process of formation of the Brazilian people arose from a Brazilian research paper applying population genetic studies, through the genetic structure, evidencing a tri-hybrid ethnicity (EUR, AFR and AMR ancestry components). An elevated mean of EUR genomic ancestry (37.1%) was identified in black Brazilian individuals^13^.

The Human Genome Project (HGP) conclusion triggered a scientific and technological advance, with the sequencing of the 3.1 billion nitrogen bases of the human genome, allowing for the identification of DNA variations, with the use of currently available tools^37^. Genome wide association studies (GWAS) have assessed the genetic basis of certain characteristics, such as susceptibility to the clinical manifestations of several diseases^27^. A large portion of GWAS studies, which seek variants associated with medical or evolutionary phenotypes, are limited to European ancestry populations. It is worth mentioning that African populations may display greater genetic variability and, therefore, present interesting variants^11,27^. Studies on Brazilian populations value the different phenotypic characteristics present in these sets, considering that most Brazilians present a significant level of African ancestry^18^. The effect of the population substructure is an effect of the differences in the proportions of European and African strata between the case and control groups, and the analysis of ancestry components aids in measuring and adjusting the population stratification in these studies^24^. In addition, considering that the subjectivity of self-reported “skin colour” classifications, such as those applied by the Brazilian census, these classifications are inadequate to describe the population structure present in mixed race populations^38^.

The types and amounts of genomic markers used to identify EUR, AFR and AMR ancestry contributions displayed different characteristics. The discrepancies between ancestry component data among the studies may be due to the differences in the number of sampled individuals and in the markers used to estimate the ancestries^12^. To solve this problem, markers with frequencies greater than or equal to 40% were chosen, increasing their ability to distinguish between ancestral populations^2,26,30,39^. Some studies point to the possibility of adequately estimating individual ancestry proportions with the use of a reduced number of markers^40^. The results, however, should be interpreted cautiously^26^. No study applied mtDNA or NRY markers to investigate ancestry contributions, due to the fact that mtDNA and NRY provide a small proportion of the genetic build of an individual^18^.

The 14 different outcomes reported in 13 articles will be discussed as follows.

SLE is considered to be a genetically (10 candidate genes) complex autoimmune disorder that exhibits diverse incidence rates according to the ancestry group and presents heterogeneous clinical and laboratory features^24^. The study developed by Barbosa *et al*.^24^ indicates that comparisons between genetic and self-reported descent in SLE patients displayed at least 30% of undeclared ancestry background, including African/Amerindian in whites and European/Amerindian in blacks. The only SLE self-declared as a black patient showed a greater degree of African ancestry (52.2%). However, the patient also presented a significant contribution of European ancestry (38.9%). Individual perception on phenotypic characteristics is imperfect, and the colour of a person’s skin cannot be properly defined^18^. Significant differences were identified in cases and controls for individuals with EUR and AFR ancestry from a broad genomic composition obtained from a series of individuals with SLE. Patients with SLE presented greater African contribution (22%) than controls (13%), whereas controls displayed 12% higher European ancestry than SLE patients^24^.

The interest in researching ancestry contributions in individuals infected with *Leishmaniachagasi* (VL),in endemic peri-urban areas of Natal city, by Ettinger *et al*.^28^ emerges from the hypothesis that some Brazilian subpopulations may be more susceptible than others to different clinical outcomes after infection^41,42^. Three mutually exclusive phenotype categories were employed: (1) VL: subjects who had either ongoing or a history of active, symptomatic VL disease; (2) DTH+: subjects with no history of documented VL who had a positive DTH skin test to Leishmania antigen; and 3)DTH−: subjects who lived in a household where 40% or more family members were infected (VL+ or DTH+). A marker located near 22q12 pointed to European ancestry in populations with different clinical phenotypes, suggesting that this region’s population may contain genes that determine the course of *L. chagasi* infection. A marker close to that locus (D22S1169) previously reported as potentially linked or associated with susceptibility to VL presented Z > 2 scores for excessive European ancestry, only for VL+ individuals (p < 0.0001). However, no clear significant difference in the total proportions of population miscegenation among the three clinical phenotype groups was noted^28^.

Concerning another infectious disease, malaria infected people were investigated for ancestry component identification. Two of malaria studies presented in this review refer to infection by the *Plasmodium* parasite^27,29^, the etiological agent of the world’s leading parasitic disease, responsible for approximately 214 million of cases a year and resulting in over 438,000 deaths^43^. A sample comprising *Plasmodium falciparum*-infected individuals in Porto Velho, Rondônia, was investigated in order to determine if variations in GYPB*S/s alleles (Glycophorin B Receptors Y) would influence susceptibility to *P. falciparum* infection in the Brazilian mixed-race population. Preliminary studies pointed to a potential association between *GPBS*+ patients and their status as *P. Falciparum*-infected, although the significance of these results was affected by the lack of a control miscegenation group, which is a potential leading factor. A miscegenation control is essential in genetic epidemiological studies carried out in Latin American populations, where large interindividual differences in the race mix are recurrent^44^. Considering this, Tarazona *et al*.^29^ measured the ancestry components between cases and controls by AIMs^29^. The authors indicate that the miscegenation indices among the studied groups did not differ significantly, and increased susceptibility to infection by this parasite is associated with the *GPBS* + variant in the Brazilian Amazon population^29^. In addition, Furini *et al*.^27^ investigated the association between *TNFA*, *INFG* and *IL10* polymorphisms and *vivax* malaria and genomic ancestry in a sample of an endemic area population in the Brazilian Amazon. According to the analysed data, *TNF, INFG* and *IL10* polymorphisms were not associated with risk or protection against this disease. However, a downward trend in the *TNFA-308* allele frequency was noted with increasing European ancestry^27^. It should be noted that Furini *et al*.^27^ and Tarazona-Santos *et al*.^29^ carried out investigations in the Brazilian Amazon, which has the highest AMR contribution (0.32) among its individuals when compared to other Brazilian regions^23^.

For sickle-cell anaemia^32^, one study was carried out in Bahia, Northeast Region, Brazil, with noteworthy results. The major genetic contribution of African ancestry was not confirmed in sickle-cell anaemia cases, which popular knowledge often claims to be restricted to black individuals. This paper refers only to the probability of an individual contracting the disease, since a person with a low proportion of African ancestry may also be affected. In selected sickle cell anaemia cases from Bahia, the observed miscegenation is in conformity with the theory that the sickle-cell disease is not only a “black disease” in Brazil, specifically in Bahia, although it is originally an African disease. Therefore, it is an eminently geographic disease, the product of a well-succeeded evolutionary strategy of humans to cope with malaria^34^.

A study on PCa also carried out in the Brazilian Northeast, assessed estimates of AFR, EUR and AMR contributions in a sample comprising 213 control-cases (104 patients and 109 paired controls) and identified a higher degree of EUR ancestry among fast developing cancer cases compared to intermediate or slow development cases. This description reinforces the understanding of the genetic basis for tumour aggressiveness among groups presenting different genetic ancestries, especially mixed-race populations, and has significant implications for interpopulation heterogeneity assessments concerning drug treatment effects^30^. It is important to mention that the authors^30^ did not discuss the environmental influence on PCa development.

Ramos *et al*.^36^ reinforced the idea of^45,46^ who cited the importance of genetic and environmental variations in the susceptibility to the development of PTL and PPROM. The variations in mothers and their babies were analysed in the state of São Paulo, as well as the discrepancies between different outcome rates for different ethnic groups and populations^4^. In the PTL subgroup assessment ancestry, EUR ancestry was higher when compared to the controls (p = 0.002), while AFR ancestry was higher in the controls when compared to PTL (p = 0.009). Higher European ancestry among PTL patients may indicate a higher risk for PTL occurrence in Southeast Region, Brazil^36^. PTL was not only associated with EUR ancestry but also with smoking, while AFR ancestry was protective. The fetal alleles *IL10-592C* (rs800872) and *IL10-819C* (rs1800871) were also associated with PTL and the maternal haplotype *TNFA-308G-238A* was protective. Maternal presence *of IL10-1082G* (rs1800896) and *TLR2A* (rs4696480) alleles increased the risk for PPROM while *TNFA-238A* (rs361525) was protective. Family history of PTL/PPROM was higher in cases, and time to delivery was influenced by *IL1B-31T* (rs1143627) and *TLR4-299G* (rs4986790)^36^.

The study of Rolim *et al*.^33^, a pioneer study in Brazil^33^, reported the relationship between genetic ancestry and PCG clinical data. This investigation showed the variations for risk and prognostic factors in a sample of children from the Brazilian Southeast. In this case, the estimates for the individual genetic ancestry components were possible when an INDEL marker was applied. A higher proportion of African INDELs was associated with increased eyeball growth and a high number of intraocular pressure control surgeries, i.e. a worse prognosis (P = 0.036). However, genetic ancestry proportion differences between the case and control groups were not significant^33^. It is important to mention that most cases of PCG are sporadic; however there is a familial autosomal recessive form (10-40%) with variable penetrance and high rate of consanguinity.

T1D, which results from the autoimmune destruction of pancreatic β (beta) cells, is a polygenic multifactorial disease that is influenced by both genetic and environmental contributing factors. Few studies have assessed T1D genetic risks in Brazil, and none considered ancestry^34^. The main locus for susceptibility to T1D traces back to class II HLA-DRB1 and HLA-DQB1 loci on chromosome 6p21^47^. Based on this fact, a Brazilian study was developed considering 1704 participants from the city of São Paulo in the Brazilian Southeast. Patients with T1D presented an average of 77% EUR, 15% AFR and 7.3% AMR, while controls, on average, presented 71% EUR, 21% AFR and 7.9% AMR. Ancestry correlations indicated that the DRB1*16 allele and -DRB107-DQB1*0201 haplotype were protective for T1D, that the DQB1*0501 allele, initially characterized as protective, was neutral, and that the haplotype DRB1*10-DQB1*0501 was protective. The correlation also confirmed that DRB1*09-DQB1*0202cause susceptibility and DRB1*0302-DQB1*0402, DRB1*10-DQB1*0501, DRB1*11-DQB1*0602 and DRB1*13-DQB1*603 present a protective effect in the population, which is similar to the effects observed in African Americans, but not in Caucasians^34^. European ancestry is the greatest contribution in Brazilians, followed by smaller African and Amerindian ancestral population contributions. The subtyping of -DQA1 and other neutral or protective DR alleles is important for identifying alleles that provide very strong protection against T1D and individuals who will not progress to the disease. These results are missing in the study, as well the influence of environmental factors^34^.

Non-syndromic oral clefts are described as the most common birth congenital disability worldwide, with a prevalence of 1.43:1,000 live births^48^. The reported prevalence among Brazilian newborns is of 0.36 and 1.54:1000 live births, with approximately 4000 new cases each year^49,50^. The investigation of the risk^35^, by the polymorphic *CRISPLD2* and *JARID2* variants and susceptibility to non-syndromic oral fissures, comprised a sample of 785 Brazilian patients. The genetic ancestry variation for each individual was considered since the results indicate a higher relation between the risk of isolated non-syndromic cleft palate only (NSCPO) and patients with high percentage of European ancestry concerning the *CRISPLD2* rs4783099 T allele^35^. The results suggest that the SNP rs4783099 in *CRISPLD2* may contribute to an increased risk of NSCPO, and the SNP rs2237138 in *JARID2* may hold a protective effect against non-syndromic cleft lip with or without cleft palate (NSCL ± P), in the Brazilian population. However, the authors emphasize that larger studies are required to validate the findings and gene-gene and gene-environment interactions should be considered in future investigations^35^. Integration of genetic and environmental risk using epigenetics, systems biology, gene expression and epidemiology will all be required to generate a synthesis for better characterizing aetiologies, access to clinical care and prevention^48^.

MS is a highly prevalent demyelinating disease in Caucasian population, in which almost 200 genes, with odds ratio (OR) ranging from 3.5 to 1.05, have already been discovered in large samples^51^. Brazilian researchers studying MS have been attempting to reach a better understanding of ancestry influence on MS prognosis through ethnic estimations. Research in genetics and pharmacogenomics may clarify the differential neurodegenerative MS progression among groups from different ancestral origins. In a recent publication, the influence of African ethnicity was marked as an unfavourable factor for all MS outcomes, although this study was only based on self-declared ancestry, not on AIMs^52^. Through the analysis of genetic ancestry estimates, the following components for patients with MS from São Paulo State were identified by Brum *et al*.^25^: AFR 12.5% and EUR 78.5%. However, these data differ from the studies carried out in Rio de Janeiro, applying a phenotypic evaluation, which described the representativeness of Afro-Brazilians as being at least 30% in MS cases^52,53^. Recent studies indicate that ethnicity should be considered for the treatment definition, particularly in admixed populations, such as the Brazilian population in South America (SA)^52^.

Frequently, the results on the description of phenotypic ethnicity versus AIM application are divergent, particularly when the samples belong to different geographic regions. This scenario can be demonstrated through NMO, which shows a peculiar rare form of central nervous system (CNS) neuroinflammatory disorder and is more frequent among Asian and AFR^25,53^. An epidemiological study carried out in SA, characterizing patients by phenotypic characteristics, such as lip thickness and nose shape, skin colour and hair texture, identified that 36.8% of patients with NMO were non-white^53^. According to a multicentric study, which included data from 17 Brazilian cities, conducted by Papais-Alvarenga *et al*.^53^, the frequency of non-whites ranged from 1.3% in Joinville (South Region) to 96.8% in Recife (Northeast Region). However, one study elected for this review, using AIMs, described the ancestry component among NMO patients from different Brazilian regions, such as: Southeast Region –(Ribeirão Preto, São Paulo and Belo Horizonte); Center-West - Goiânia and Northeast Region - Recife, as 20.5% AFR and 68.7% EUR^25^. In both studies, which assessed ancestry by applying different methodologies^25,53^, the results were very dissimilar, thus reaffirming that the interviewer’s assessment, without genetic-based indicators, leads to limited results when based on ethnicity/skin colour classifications.

Researchers must recognize the potential influence of their personal values^10^, including ethnocentrism. From this idea, it is uncovered that study participants and/or researchers have their own consciousness, “influenced” by prejudice, social stigma and culture regarding data collection for health research regarding “race”. Therefore, different strategies should be combined, such as the interviewee’s self-categorization, with both open and closed questions (predefined categories), the interviewee’s classification by the interviewer’s assignment (predefined categories), and the inclusion of open and closed questions regarding ethnicity^14^.

Still in the neurology field, Benedet *et al*.^26^ followed a cohort of patients with AD from the metropolitan area of Brazil’s Federal District for approximately two years. Their results pointed to mean genetic contributions of 56.8% from Europeans, 29.3% from Africans and 13.9% from Native Americans, comparable to ancestry percentages found in other parts of Brazil, with around 60–75% from Iberian whites, 10–30% from Western Africans and 5–20% from Native Americans^21,54^. The proportions of EUR and AFR ancestries were significantly higher, while the proportion of AMR ancestry was lower for patients with AD. The average content for AMR genetic ancestry was 3-fold lower (5.6 vs. 16.2%, p < 0.001) than the corresponding average content, indicating that the Amerindian allelic architecture could confer protection against AD development, however, they suggest that some caution should be taken in the interpretation of their results^26^. Benedet *et al*.^26^ emphasized that AD depends on modifiable factors (cultural, educational and social aspects) and on non-modifiable factors (e.g. genetic architecture- e4allele of Apo E).

Bernadez-Pereira *et al*.^31^ investigated the genomic ancestry and hemodynamic patterns of Brazilian patients with HF in São Paulo. They emphasize the need for caution when using self-declared race as an ancestry marker or extrapolating the results from a mixed population to another. A quantitative method was applied for characterizing ancestry and hemodynamic patterns in Brazilian patients with HF^31^. The EUR, AFR and AMR ancestry distributions were of 61%, 29% and 6%, respectively. Once again, considerable discrepancy between self-reported ancestry percentage and quantitative measures among patients was noted. Thus, self-reported skin colour was not useful for inferring hemodynamic profiles in HF cases. AFR genetic ancestry was related to the worst diastolic function parameters (r = 0.197, p < 0.01), while AMR ancestry was correlated with a worse ventricular contractility pattern (r = 0.109, p < 0.05). Ethnic differences in cardiovascular events are mediated by genetic factors which determine the severity of the disease and therapy responses in HF cases^55^. Previous reports on HF mortality and hospitalization rates are higher among African Americans compared to whites^56^. However, these studies may be influenced by various misleading factors due to intercultural, educational and social aspects.

In the course of this systematic review, some limitations of the assessed studies were identified. These include the small number of controls and/or cases^27,30,31^, as well as the small number of AIMs^26,30^. External validity may also be compromised, since data on genetic ancestry may vary between regions^31^, so they cannot be extrapolated to the five Brazilian regions.

In conclusion, the results showed that: higher EUR ancestry was identified in cases of PTL, T1D, non-syndromic oral fissure and aggressive prognoses in patients with prostate cancer; on the other hand, the highest AFR ancestry component was observed in SLE and PCG evidencing worse prognoses; AMR ancestry may be a protective factor in the development of AD; the worse hemodynamic parameters in HF cases were identified among individuals presenting higher AMR and AFR ancestry. In general, these results suggest that studies that apply the race/skin colour/ethnicity variable in Brazil in the absence of a quantitative method when describing ancestry are subject to preconceived ideas. Thus, participating individuals as well as the observers (researchers) are not exempt from mischaracterizing the object of study-ancestrality. Phenotypic characteristics supported by genetic factors, such as hair colour and texture, nose or lip thickness or skin colour, when compared to genetic ancestry EUR, AFR and AMR components, should not be ignored, but instead considered with caution when either worse prognoses, protective effects or frequency outcomes are described.

It is worth mentioning at this point that heritability estimates are very important because the heritability values show what proportion of variation in a given phenotype is due to genes and environment ranging from 0 (no genetic contribution) to 1 (all differences on a trait reflect genetic variation). Therefore, environment has an important contribution to various diseases presented here, especially in Brazil which is a country of continental size with big social and economic inequalities. Furthermore, we have to point out that epigenomic variation can exhibit distinct responses to environmental stresses, contributing to significant proportion of the altered susceptibility to human diseases^57^.

As a way to put information in an accurate perspective, we included a list of supplementary references on heritability for the traits under analysis in this review, but with data from other countries, so that the genetic and environmental contributions can be evaluated by the reader.

## Material and Methods

### Information sources and research strategy

The information sources and research strategy were constructed using the PICO strategy (P-Population: A ancestry/group of people; I-Intervention: Genetic testing using ancestry informational markers, O-Outcome: Brazilian ancestry used to construct the research question and search for evidence)^58^. The MESH (Medical Subject Headings) controlled vocabulary database was applied.

The process concerning study identification for incorporation into this review was carried out by a search at the MEDLINE (Medical Literature Analysis and Retrieval System Online), via PubMeb, and LILACS (Scientific and Technical Literature of Latin America and the Caribbean/VHL - Virtual Health Library) electronic databases. The descriptors were obtained from the DeCS (Health Sciences Descriptors) and MeSH (Medical Subject Headings) listings. Portuguese and/or English terms were used, as well as truncation symbols (dollar sign or asterisk) to search for words with the same linguistic root, increasing the chances of detecting a greater number of papers. The search was restricted from 2005 to 2017, using the following terms:ancestry[tiab] OR european continental ancestry group [MeSH] OR white* [Tiab] OR caucas* Race [tiab] OR african continental ancestry group [Mesh] AND ds DNA [tiab] OR deoxyribonucleic acid [Tiab] OR genetic markers [Mesh] OR Genetic Marker [tiab] OR DNA markers [tiab] OR marker chromosome [tiab] OR genetics [Mesh] AND Brazil [Mesh] OR geographical locations [tiab] OR locations americas south [tiab] OR America Brazil [tiab] OR Brazilians [tiab].

### Eligibility Criteria

Original papers, published between 2005 and 2017 that aimed to identify the genetic ancestry of Brazilian populational groups presenting disease cases by applying genomic ancestry markers were included. Editorial works, case studies, reviews, pilot studies, series studies, theses or dissertations were not included, as well as papers whose samples did not fully comprise Brazilian individuals. Additionally, studies that only used self-declared or visually identified ancestry were excluded. This review was limited to the Brazilian genetic composition context and only studies carried out in Brazil were selected.

### Paper selection and data collection process

Papers identified in more than one database were considered only once. Full papers were obtained when insufficient information was available for a detailed paper title and abstract analysis was noted. Subsequently, the complete texts which met the eligibility criteria were stored and catalogued in a digitalised bank. Each study received an identification number, created from a combination of the first author and publication year. The following details were highlighted: Detail on participant individuals, including healthy subjects and patient samples (cases); Brazilian region where the participants were selected from; results of EUR, AFR and AMR ancestry components, and their influence on the assessed health outcomes.

### Study quality evaluation

**Figure 2:**
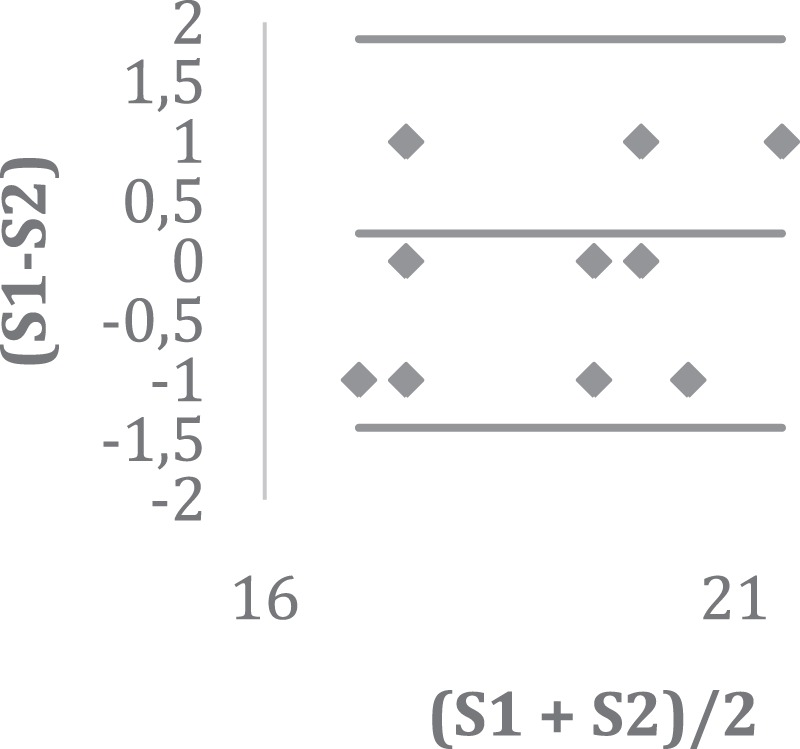
Conformity between STROBE (Strengthening the Reporting of Observational Studies in Epidemiology) assessment results; S1 = researcher 1/STROBE 1; S2 = researcher 2/STROBE.

Regarding study quality evaluation, all papers were submitted to the STROBE evaluation method (Strengthening the Reporting of Observational Studies in Epidemiology), for observational studies in epidemiology^59^. It should be noted that this study aimed to identify the pertinence of the selected studies, so that the panel generated by their information could easily display the quality of each study. The questions were treated in a dichotomous manner, by two specialists (FSCP and RMG), with maximum paper score of 22, equivalent to the number of items presented in the STROBE instrument. Finally, result conformity was evaluated by comparing the assessments of both experts (Table 3), using the intraclass correlation coefficient (ICC) and the Shrout scale, which can be interpreted as follows: CCI ≤ 0.4 poor correlation; 0.4 ≤ CCI ≤ 0.75 satisfactory to good; CCI > 0.75excellent. Additionally, the Altman-Bland diagram was used to assess the worsening or improvement of the conformity trend between the extreme values of the ranking (i.e., the worst and the best published studies), as well as bias insertion by some of the reviewers (Fig. 2).

**Table 3:** STROBE assessment.

Reference	STROBE 1	STROBE 2	(S1-S2)	(S1 + S2)/2	(S2-S1)	Mean Dif.	LI	LS
Benedet *et al*.^26^	21	20	1	20.5	−1	0.23	−1.40	1.86
Brum *et al*.^25^	18	17	1	17.5	1	0.23	−1.40	1.86
Ettinger*et al*.^28^	18	17	1	17.5	1	0.23	−1.40	1.86
Tarazona-Santos *et al*.^29^	18	17	1	17.5	−1	0.23	−1.40	1.86
Barbosa *et al*.^24^	20	20	0	20	0	0.23	−1.40	1.86
Furini *et al*.^27^	17	18	−1	17.5	0	0.23	−1.40	1.86
Gomes *et al*.^34^	20	20	0	20	1	0.23	−1.40	1.86
Oliveira *et al*.^30^	20	20	0	20	1	0.23	−1.40	1.86
Messetti*et al*.^35^	19	20	−1	19.5	−1	0.23	−1.40	1.86
Bernardez-Pereira *et al*.^31^	20	19	1	19.5	0	0.23	−1.40	1.86
Nascimento *et al*.^32^	17	18	−1	17.5	0	0.23	−1.40	1.86
Rolim *et al*.^33^	17	17	0	17	−1	0.23	−1.40	1.86
Ramos *et al*.^36^	22	21	1	21.5	1	0.23	−1.40	1.86
Mean	19.00	18.77	0.23	18.88				
Deviation	1.63	1.48	0.83					
LL			−1.40					
UL			1.86					

LL = Lower limit; LS = Upper Limit; Mean Dif = Mean Differences; STROBE^59^ = Strengthening the Reporting of Observational Studies in Epidemiology; S1 = researcher 1/STROBE 1; S2 = researcher2/STROBE 2.

## Supplementary information

Supplementary references
