# Author Correction: A systematic literature review on the European, African and Amerindian genetic ancestry components on Brazilian health outcomes

**DOI:** 10.1038/s41598-020-64606-z

**Published:** 2020-05-01

**Authors:** Fabiana dos Santos Carolino Firmo Pereira, Raphael Mendonça Guimarães, Alexandre Ramos Lucidi, Doralina Guimarães Brum, Carmen Lucia Antão Paiva, Regina Maria Papais Alvarenga

**Affiliations:** 1 Department of Neurology, Graduate Program in Neurology (PPGneURO) – Universidade Federal do Estado do Rio de Janeiro, UNIRIO, Rio de Janeiro, RJ 20270-004 Brazil; 2 Research Center for Population and Public Policies Studies, Rio de Janeiro, Brazil. Oswaldo Cruz Foundation, Rio de Janeiro, RJ 21040-900 Brazil; 3 Departamento de Neurologia, Psicologia e Psiquiatria, Faculdade de Medicina de Botucatu, Universidade Estadual Paulista -UNESP, Botucatu, São Paulo Brazil; 4 Department of Genetics and Molecular Biology, UNIRIO, Rio de Janeiro, 20211-010 RJ Brazil; 5 Graduate Program in Molecular and Cell Biology (PPGBMC). UNIRIO, Rio de Janeiro, RJ 20211-010 Brazil

Correction to: *Scientific Reports* 10.1038/s41598-019-45081-7, published online 20 June 2019

This Article contains an error in the Discussion section.

“An epidemiological study carried out in SA, characterizing patients by phenotypic characteristics, such as lip thickness and nose shape, skin colour and hair texture, identified that 36.8% of patients with NMO were non-white^53^.”

should read:

“An epidemiological study carried out in SA, characterizing patients by phenotypic characteristics, such as lip thickness and nose shape, skin colour and hair texture, identified that 53.1% of patients with NMO were non-white^53^.”

