# Supplementary material for: A systematic literature review on the European, African and Amerindian genetic ancestry components on Brazilian health outcomes
Source: Sci Rep. 2019 Jun 20;9:8874. doi: 10.1038/s41598-019-45081-7 (PMC6586659; doi:10.1038/s41598-019-45081-7)
Supplement: Supplementary file 1 — Supplementary references [file 41598_2019_45081_MOESM1_ESM.pdf]

A systematic literature review on the European, African and Amerindian genetic ancestry components on Brazilian health outcomes

Fabiana dos Santos Carolino Firmo Pereira[1,*], Raphael Mendonça Guimarães[2], Alexandre Ramos Lucidi[1], Doralina Guimarães Brum[3], Carmen Lucia Antão Paiva[1,4,5], Regina Maria Papais Alvarenga[1]

[1]Department of Neurology. Graduate Program in Neurology (PPGNEURO) – Universidade Federal do Estado do Rio de Janeiro, UNIRIO, Rio de Janeiro – RJ, 20270-004, Brazil.

[2]Research Center for Population and Public Policies Studies, Rio de Janeiro, Brazil. Oswaldo Cruz Foundation. Rio de Janeiro, RJ, 21040-900, Brazil.

[3] Departamento de Neurologia, Psicologia e Psiquiatria, Faculdade de Medicina de Botucatu, Universidade Estadual Paulista -UNESP, Botucatu, São Paulo, Brazil.

[4]Department of Genetics and Molecular Biology, UNIRIO

[5]Graduate Program in Molecular and Cell Biology (PPGBMC). UNIRIO. Rio de Janeiro-RJ, 20211-010, Brazil.

*Correspondence: email address: fabiana.pereira@unirio.br

Supplementary references

Brito, L.A.*et al.* IRF6 is a risk factor for nonsyndromic cleft lip in the Brazilian population. Am J Med Genet A. 158A, 2170-5(2012).
Browning, S.R, Browning, B.L. Population structure can inflate SNP-based heritability estimates. Am J Hum Genet. 89,191-3, author reply 193-5 (2011).
Campion, D. *et al*. Alzheimer disease: modeling an Aβ-centered biological network. Mol Psychiatry. 21, 861-71 (2016).
Cheng, S. *et al*. Left ventricular mechanical function: clinical correlates, heritability, and association with parental heart failure. Eur J Heart Fail. 17,44-50 (2015).
Cotsapas, C., Mitrovic, M., Hafler, D. Multiple sclerosis. Handb Clin Neurol. 148,723-730 (2018).
Darst, B.F. *et al*. Heritability of cognitive traits among siblings with a parental history of Alzheimer's disease. J Alzheimers Dis. 45, 1149-55 (2015).
Frade, A.F. Visceral leishmaniasi and genetic susceptibility. Revista da Biologia. 6b, 22-25(2011)

Grasso, M.G., Frontali, M., Bernardi, S., Pantano, P. *et al*. Multifactorial inheritance and recurrence risks of multiple sclerosis in Italian patients. Neuroepidemiology. 8,300-7 (1989).
Grosen, D. *et al*. Risk of oral clefts in twins. Epidemiology. 22,313-9 (2011).
Gusev, A. *et al*. Atlas of prostate cancer heritability in European and African-American men pinpoints tissue-specific regulation. Nat Commun.7,10979 (2016).
Hoffmann, T.J. *et al*. A large multiethnic genome-wide association study of prostate cancer identifies novel risk variants and substantial ethnic differences. Cancer Discov. 5, 878-91 (2015).
Kano, F. S. *et al.* The Presence, Persistence and Functional Properties of *Plasmodium vivax* Duffy Binding Protein II Antibodies Are Influenced by HLA Class II Allelic Variants. PLoS neglected tropical diseases, 10, e0005177 (2016).
Kim, N.R. *et al.* Heritabilities of intraocular pressure in the population of Korea: the Korean National Health and Nutrition Examination Survey 2008-2009. JAMA Ophthalmol.132, 278-85 (2014).
Lee, S. H., Wray, N. R., Goddard, M. E., & Visscher, P. M. Estimating missing heritability for disease from genome-wide association studies. American journal of human genetics. 88, 294–305 (2011).
Lichtenstein, P. *et al.* Environmental and heritable factors in the causation of cancer-analyses of cohorts of twins from Sweden, Denmark, and Finland. N Engl J Med. 343, 78-85 (2000).

Loucoubar, C. *et al.* Impact of changing drug treatment and malaria endemicity on the heritability of malaria phenotypes in a longitudinal family-based cohort study. PLoS One. 6, e26364 (2011).
Monte, E., Vondriska, T.M. Epigenomes: the missing heritability in human cardiovascular disease? Proteomics Clin Appl. 8,480-7(2014).
O'Gorman, C., Lin, R., Stankovich, J., Broadley, S.A. Modelling genetic susceptibility to multiple sclerosis with family data. Neuroepidemiology. 40, 1-12. (2013).
Park, S. *et al*. Adjusting heterogeneous ascertainment bias for genetic association analysis with extended families. BMC Med Genet.16,62(2015).

Phimpraphi, W. *et al.* Heritability of P. *falciparum* and P. *vivax* malaria in a Karen population in Thailand. PloS One, 3, e3887 (2008).
Ridge, P;G., Mukherjee, S., Crane, P.K, Kauwe, J.S. Alzheimer’s Disease Genetics Consortium. Alzheimer's disease: analyzing the missing heritability. PLoS One.8, e79771 (2013).
Szulkin, R. *et al*. Estimating Heritability of Prostate Cancer-Specific Survival Using Population-Based Registers. Prostate. 77,900-907 (2017).
Wang, J. *et al*. Systemic lupus erythematosus: a genetic epidemiology study of 695 patients from China. Arch Dermatol Res. 298, 485-91(2007).
Williams, D.R. Epidemiological and geographic factors in diabetes. Eye (Lond).7,202-4 (1993).
Yates, S.C. *et al*. The effects of two polymorphisms on $p21^{cip1}$ function and their association with Alzheimer's disease in a population of European descent. PLoS One. 10, e0114050 (2015).
Yendry, S.B &, Horacio, A. C. G. The socio-environmental determinants of malaria in the town of Matina in Costa Rica. Revista Costarricense de Salud Pública, 21, 50 -57(2012).
